# TRK Inhibition with Entrectinib in Metastatic Salivary Secretory Carcinoma (SC): A Case Report

**DOI:** 10.3390/curroncol29060314

**Published:** 2022-05-31

**Authors:** Matthew S. Ernst, John T. Lysack, Martin D. Hyrcza, Shamir P. Chandarana, Desiree Hao

**Affiliations:** 1 Tom Baker Cancer Center, Department of Oncology, Cumming School of Medicine, University of Calgary, Calgary, AB T2N 4N2, Canada; matthew.ernst@albertahealthservices.ca; 2 Division of Head and Neck Imaging, Department of Radiology, Cumming School of Medicine, University of Calgary, Calgary, AB T2N 4N1, Canada; john.lysack@albertahealthservices.ca; 3 Department of Pathology and Laboratory Medicine, Arnie Charbonneau Cancer Institute, University of Calgary, Calgary, AB T2N 2T9, Canada; martin.hyrcza@albertaprecisionlabs.ca; 4 Section of Otolaryngology, Department of Surgery, University of Calgary, Calgary, AB T2N 2T9, Canada; shamir.chandarana@albertahealthservices.ca; 5 Ohlson Research Initiative, Arnie Charbonneau Research Institute, Cumming School of Medicine, University of Calgary, Calgary, AB T2N 4Z6, Canada

**Keywords:** secretory carcinoma, NTRK, entrectinib, larotrectinib, targeted therapy, molecular testing

## Abstract

NTRK gene fusions are rare oncogenic driver mutations that can be found in a broad range of neoplasms. In secretory carcinoma (SC), *ETV6-NTRK3* gene fusion is seen in a majority of the cases and represents a druggable target for patients with advanced disease in the absence of a currently accepted standard of care. In our case, we describe a patient with recurrent, metastatic SC treated with first line entrectinib with clinically meaningful, durable ongoing response after 49 months. The patient experienced grade 1 fatigue, dysgeusia, skin sensitivity, arthralgias, an increase in serum creatinine, and weight-gain as well as grade 2 hypotension which resolved after a dose reduction. Entrectinib is a well-tolerated treatment with the potential for durable responses and TRK inhibition should be considered the standard of care in SC and other *NTRK* gene fusion-positive advanced neoplasms without acceptable alternative treatment options.

## 1. Introduction

The recognition of *NTRK* gene fusions as rare but important driver mutations has led to the development of therapeutic tropomyosin receptor kinase (TRK) blocking agents. The TRK family plays an important role in the development and homeostatic regulation of the nervous system [1]. The genes *NTRK1*, *NTRK2*, and *NTRK3* encode receptors TRKA, TRKB, and TRKC, respectively. TRK oncogenes are formed through chromosomal rearrangements and gene fusion events that result in constitutive activation through altered neurotrophin binding specificity or alter downstream signaling independent of neurotrophin interaction. *NTRK1* and *NTRK3* gene fusions have most commonly been identified; however, *NTRK2* fusions have also been demonstrated [2,3]. Since the first discovery of an *NTRK* fusion gene in 1983, more than 80 TRK fusion partners have been identified in a broad range of tumor types, among pediatric and adult populations [1]. The frequency with which *NTRK* gene fusions can be found varies substantially between tumor groups. Certain rare malignancies such as salivary secretory carcinoma (SC), congenital fibrosarcoma, or secretory carcinoma of the breast are typically enriched for *NTRK* gene fusions with a prevalence >80%. More common malignancies such as colorectal, lung, pancreatic, or breast cancers, in contrast, have a low prevalence of *NTRK* gene fusions, often less than 1% [2,4,5].

Salivary SC is an uncommon neoplasm characterized by the frequent presence of an *ETV6-NTRK3* gene fusion in addition to distinct histologic features. Systemic treatments for recurrent, metastatic salivary SC have not been standardized. SC was designated a separate entity from acinic cell carcinomas (AciCC), which are morphologically distinct from all other known salivary tumors. While SC and conventional AciCC have similar morphologic features, the major distinguishing characteristics of SC are the absence of acinar cells and the presence of a t(12;15) (p13;q25) *ETV6-NTRK3* chromosomal rearrangement [6,7]. SC most closely resembles secretory carcinoma of the breast, another rare neoplasm that frequently harbors a *ETV6-NTRK3* fusion [6]. In this report we describe a case of recurrent, metastatic salivary SC treated with entrectinib, a potent TRK inhibitor, and intend to highlight the therapeutic application which led to a clinically meaningful and durable ongoing response.

## 2. Case Presentation

**Figure 1:**
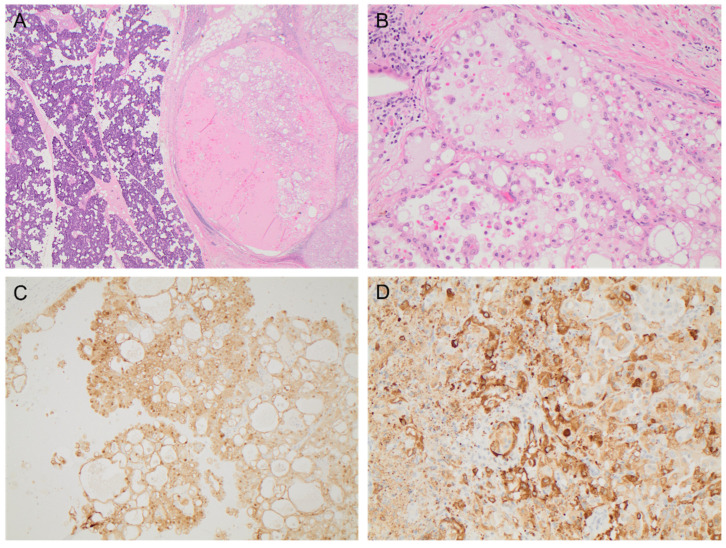
The resected primary parotid mass demonstrated features of SC with (**A**) a well-circumscribed tumor characterized by a vacuolated amphiphilic epithelium and papillary-cystic architecture (H&E, ×20), (**B**) abundant solid areas with microcystic spaces (H&E, 200×), and diffuse strong immunohistochemistry staining for (**C**) S100 (×100) and (**D**) mammaglobin (×200).

A 59-year-old male lifetime non-smoker, with hypertension, who was otherwise well, presented with a 6-month history of an enlarging right pre-auricular mass. A fine-needle aspirate (FNA) of the mass raised suspicion for AciCC or mucoepidermoid carcinoma. A CT of the neck confirmed the presence of a 1.6 × 1.2 × 1.8 cm soft tissue mass involving the superior margin of the superficial lobe of the right parotid gland in close proximity to the facial nerve and extending into the overlying fat. No pathologically enlarged or suspicious lymph nodes were present in the neck on either side. Therefore, the parotid tumor was clinical stage III (cT3N0M0) at diagnosis by the American Joint Committee on Cancer (AJCC) 8th edition cancer staging manual [8]. It was felt the risk of microscopic lymph node involvement was low since the FNA had suggested AciCC as the underlying pathology and therefore, the patient underwent a right parotidectomy without a comprehensive lymph node dissection. The final pathology revealed a well circumscribed tumor, with vacuolated amphiphilic epithelium and papillary-cystic architecture (Figure 1). Immunohistochemistry showed the tumor cells were strongly positive for S100, vimentin, mammaglobin, and cytokeratin 19, characteristic features of SC but not AciCC or mucoepidermoid carcinoma. An *ETV6* rearrangement was detected by fluorescence in situ hybridization (FISH) and next generation sequencing (NGS) with fusion plex solid tumor kit identified an *ETV6-NTRK3* gene fusion, which confirmed the diagnosis of SC. Two lymph nodes were resected with the primary mass and were negative for malignancy. Adjuvant radiation therapy was offered to the patient after the final pathology was known and due to a close resection margin of 0.2 cm; however, the patient declined in favor of observation.

**Figure 2:**
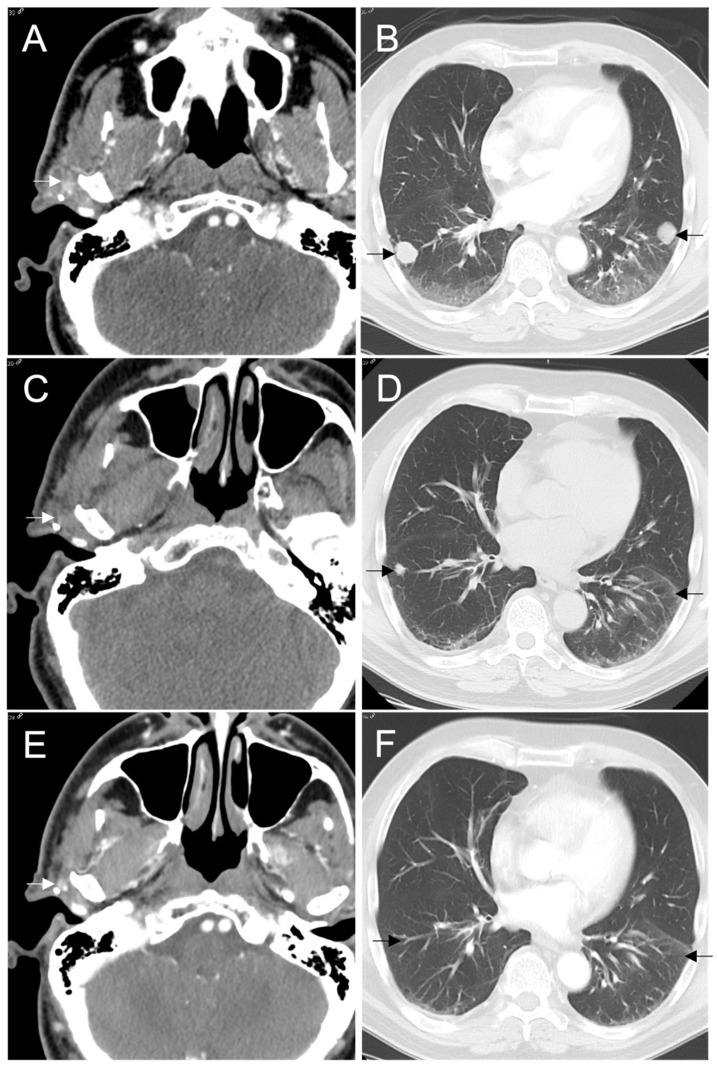
CT shows (**A**) a recurrent mass in right parotid resection bed measuring 1.0 × 1.2 cm and (**B**) index right and left lung nodules measuring 2.1 × 1.9 cm and 2.0 × 1.9 cm, respectively, at baseline prior to beginning entrectinib. A CT was performed without contrast after 4 weeks of therapy due to transient renal dysfunction, therefore, (**C**) the right parotid mass is difficult to appreciate but measured 1.1 × 0.6 cm and (**D**) the index right and left lung nodules measured 0.4 and 0.5 cm. The (**E**) right parotid mass and (**F**) index pulmonary nodules continued to decrease in size and are no longer apparent on CT at 46 months. Several tiny pulmonary nodules remain stable.

The patient developed a right pre-auricular, subdermal nodule five years later. Repeat CT of the neck and chest confirmed the presence of 0.9 × 0.9 cm nodule anterior to the right tragus in the parotid resection bed as well as multiple, bilateral pulmonary nodules measuring up to 2.1 × 1.9 cm in the right lower lobe (RLL) and 2.0 × 1.9 cm in the left lower lobe index nodules. Biopsies of the pre-auricular nodule and RLL nodule confirmed recurrent metastatic SC and the patient was started on entrectinib at 600 mg daily. The patient had a partial response after only 4 weeks and the pre-auricular mass was no longer identifiable after 19 weeks of treatment. The index pulmonary nodules and several tiny pulmonary nodules are no longer apparent on CT, and the multiple remaining tiny pulmonary nodules remain stable at 46 months follow-up (Figure 2). Our patient tolerated entrectinib well. The only side effects reported were grade 1 fatigue, dysgeusia, skin sensitivity, arthralgias, increase in serum creatinine, weight-gain, and grade 2 hypotension resulting in two syncopal events. The dose was reduced to 400 mg daily after 6 weeks and resulted in a significant improvement in energy, skin sensitivity, arthralgias and hypotension over several weeks with no further adverse events. Grade 1 dysgeusia persisted on treatment with a partial improvement after dose reduction and creatinine gradually returned to baseline over approximately 10 months.

## 3. Discussion

Our patient was the first to have been treated through compassionate access with entrectinib in Canada. This case exemplifies the use of targeted TRK inhibition as a well-tolerated treatment achieving a rapid and durable response in an orphan disease, SC, for which there is no standard systemic therapy. Identifying *NTRK* gene fusions in tumor tissues with low prevalence and equally, the rarity of *NTRK* fusions among common cancer types, presents a challenge. Targeting these novel fusions can have significant, dramatic, anti-tumor effects, therefore, adopting standard algorithms for tumors to be tested is critical.

Historically FISH and RT-PCR have been used to detect *NTRK* chromosomal alterations. FISH requires three probes—one each for *NTRK1/2/3* and has limited ability to identify the 5′ fusion partner. Similarly, RT-PCR is a widely available technique but has limited capacity for multiplex analysis. These methods, therefore, may be useful to confirm the presence of specific *NTRK* fusions that occur with high frequency among rare tumors such as salivary SC, SC of the breast, or congenital fibrosarcoma. Immunohistochemistry (IHC) is a relatively inexpensive, more widely available method of detection using several commercially available antibodies. The presence of TRK by IHC suggests an *NTRK* fusion; however, this is not diagnostic and requires confirmation. Both RNA and DNA NGS allow the detection of not only the presence of an *NTRK* gene fusion, but also the fusion partner. In a minority of cases, the driver mutation in SC has been identified to be *ETV6-RET* or *ETV6-MET* gene rearrangements and therefore provide distinct pharmacologic targets for which RET or MET targeted therapies may be more appropriate [9,10]. The cost, time, and bioinformatics experience required to operate NGS is generally prohibitive for its use as a screening platform in entities with low prevalence. Canadian consensus guidelines regarding biomarker testing and treatment of *NTRK* fusion cancers in five tumor types (thyroid carcinoma, colorectal carcinoma, non-small cell lung carcinoma, soft tissue sarcoma, and salivary gland carcinoma) were published in February 2021 [11]. This is an important step in addressing the unmet need of identifying and providing satisfactory treatment options to patients with *NTRK*-fusion positive tumors, as in the case described. At present, using IHC as a screening tool followed by a confirmatory sequencing platform is a rational approach to balance assay limitations and resource utilization [12,13,14,15].

In 2018, larotrectinib became the first TRK inhibitor to receive tumor-agnostic approval from the FDA based on pooled phase 1 and 2 data from SCOUT, NAVIGATE, and LOXO-14001 trials for locally advanced or metastatic, *NTRK* mutation positive tumors of multiple histologic tissues [16]. In an updated, pooled analysis, 121 of 153 patients (79%, CI 72–85%) had an objective response. Among the 20 patients included in the *NTRK* mutation positive salivary gland cohort, 18/20 (90%, CI 68–99%) had a complete or partial response. A 2021 updated abstract reveals a Median PFS of 35.4 months (CI 23.4–55.7 months) and OS was not yet reached with a median follow-up of 22.3 months. This is a clinically meaningful response in a disease site for which no previous standard of care has been established [17,18].

Entrectinib, a TRK inhibitor with additional activity against *ALK* and *ROS1* arrangements, gained tumor agnostic FDA approval in 2019 for advanced NTRK positive tumors based on pooled phase 1 and 2 interim data from STARTRK-1, STARTRK-2 and ALKA-372-001 [19,20]. An integrative analysis of the three phase 1/2 studies was published in 2020 which demonstrated an objective response rate of 57% (95%, CI 43.2–70.8%) and median duration of response of 10 months (95% CI 7.1 months-NE) in 54 patients with advanced TRK positive malignancies treated with entrectinib. Of note, among 12 patients included with CNS metastatic disease, six (50%) had a partial response [21]. The latest update of the STARTRK-1, STARTRK-2 and ALKA-372-001 studies continues to show meaningful and durable responses: among 121 adult patients with *NTRK*-fusion positive tumors the response rate was 61.2%, including 15.7% complete responses, and the median progression free survival was 13.8 months (95% CI 10.1–19.9) [22].

Our case illustrates that TRK targeted therapy is a very tolerable alternative to traditional chemotherapy and immunotherapy in patients with an amenable alteration [23,24,25]. In the updated pooled safety assessment, entrectinib was discontinued due to treatment-related adverse events in 6.5% of patients [22]. Dose reductions and interruptions for toxicity were more common, occurring in 25.4% and 33.7%, respectively. The majority of treatment related adverse events (TRAEs) were nonserious, grades 1–3, and were reversible or resolved with dose reductions. Grade ≥ 3 TRAEs were reported in 41.5% [21]. The most common grade 3 or 4 treatment related adverse events were increased weight in 8.3%, anemia in 5.2% and fatigue in 4.7% of patients [21]. In the described case, the patient experienced grade 2 hypotension, which led to a dose reduction early on; however, thereafter, tolerated entrectinib well and achieved a durable response with no further dose adjustments.

Health Canada approved Larotrectinib in 2019 for the treatment of adult and pediatric patients with solid metastases that (1) have an *NTRK* gene fusion without a known acquired resistance mutation (2) are metastatic or where surgical resection is likely to result in severe morbidity and (3) have no satisfactory treatment options [26]. Similarly, entrectinib gained Health Canada approval in 2020 for the treatment of adult patients with (1) unresectable locally advanced or metastatic extracranial solid tumors, including brain tumors (2) have *NTRK* gene fusion without a known acquired resistance mutation and (3) have no satisfactory treatment options [27].

## 4. Conclusions

Identifying gene fusions in tumor tissues with low prevalence is a challenge that is not unique to *NTRK*-fusions but underscores the necessity of developing rapid and cost-effective platforms for detecting uncommon, clinically significant and druggable gene rearrangements as other oncogenic drivers are identified across diverse tumor types. Incorporating these testing methods into routine practice will help create capacity for development of drugs for tumors with rare actionable mutations. For SC, in the absence of evidence to support the efficacy of chemotherapy and immunotherapy, targeted TRK inhibition should be considered the standard of care. A tumor-agnostic approach to drug approval for tumors harboring rare gene-fusions seems a reasonable approach given the excellent response rates and tolerability of this class of agents.

## Data Availability

No new data were created or analyzed in this study. Data sharing is not applicable to this article.
